# Using salamanders as model taxa to understand vertebrate feeding constraints during the late Devonian water-to-land transition

**DOI:** 10.1098/rstb.2022.0541

**Published:** 2023-12-04

**Authors:** Daniel Schwarz, Egon Heiss, Todd W. Pierson, Nicolai Konow, Rainer R. Schoch

**Affiliations:** ^1^ Department of Palaeontology, State Museum of Natural History Stuttgart, Rosenstein 1, 70191 Stuttgart, Germany; ^2^ Institute of Zoology and Evolutionary Research, Friedrich Schiller University Jena, Erbertstrasse 1, 07743 Jena, Germany; ^3^ Department of Ecology, Evolution, and Organismal Biology, Kennesaw State University, Kennesaw, GA 30144, USA; ^4^ Department of Biological Sciences, University of Massachusetts Lowell, 198 Riverside Street, Lowell, MA 01854, USA; ^5^ Institute for Biology, Department of Palaeontology, University of Hohenheim, Wollgrasweg 23, 70599 Stuttgart, Germany

**Keywords:** amphibians, vertebrate land invasion, nutrition, intraoral food processing, heterochrony, early tetrapods

## Abstract

The vertebrate water-to-land transition and the rise of tetrapods brought about fundamental changes for the groups undergoing these evolutionary changes (i.e. stem and early tetrapods). These groups were forced to adapt to new conditions, including the distinct physical properties of water and air, requiring fundamental changes in anatomy. Nutrition (or feeding) was one of the prime physiological processes these vertebrates had to successfully adjust to change from aquatic to terrestrial life. The basal gnathostome feeding mode involves either jaw prehension or using water flows to aid in ingestion, transportation and food orientation. Meanwhile, processing was limited primarily to simple chewing bites. However, given their comparatively massive and relatively inflexible hyobranchial system (compared to the more muscular tongue of many tetrapods), it remains fraught with speculation how stem and early tetrapods managed to feed in both media. Here, we explore ontogenetic water-to-land transitions of salamanders as functional analogues to model potential changes in the feeding behaviour of stem and early tetrapods. Our data suggest two scenarios for terrestrial feeding in stem and early tetrapods as well as the presence of complex chewing behaviours, including excursions of the jaw in more than one dimension during early developmental stages. Our results demonstrate that terrestrial feeding may have been possible before flexible tongues evolved.

This article is part of the theme issue ‘Food processing and nutritional assimilation in animals’.

## 1. Why study the evolution of vertebrate feeding?

**Figure 1.**
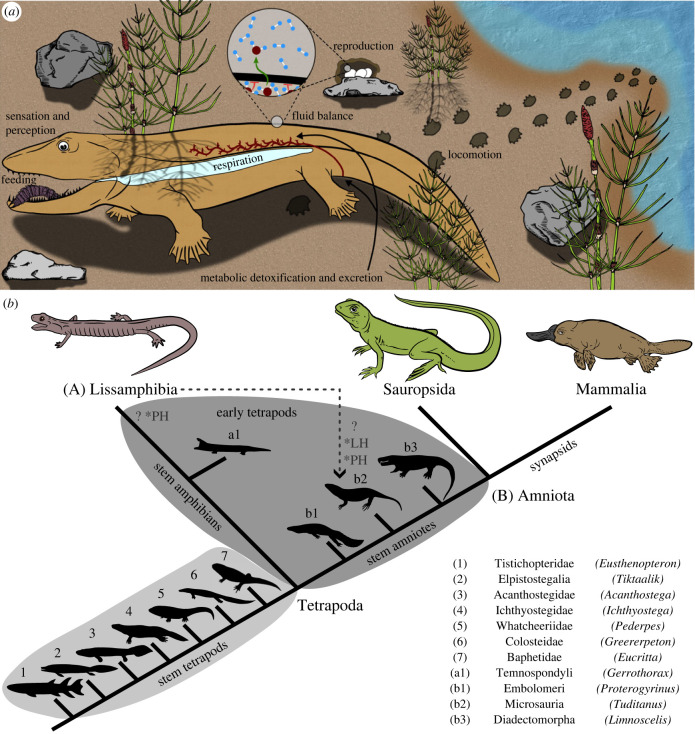
Exemplary features of terrestrialization and simplified tetrapodomorph phylogeny. (*a*) A hypothetical early tetrapodomorph displaying exemplary features required for transitioning to a permanent life on land. Traits that must adapt to terrestrial life include feeding, fluid balance (homeostasis of the water content), locomotion, metabolic detoxification and excretion, reproduction, respiration, as well as sensation and perception (gathering and processing environmental information). Feeding had to adapt to new conditions, including the physical properties of the surrounding air and potentially new prey characteristics, including the arthropod exoskeleton. Early tetrapods had to evolve strategies to maintain their fluid balance and allow for reproduction on land to allow for water-content homeostasis. Metabolic detoxification and excretion had to proceed with limited water supplies to reduce further water loss. Locomotion and the musculoskeletal system had to adapt to allow the body weight to be carried against gravity. Lung and skin respiration had to replace the oxygen uptake from gill respiration, and sensation and perception also had to adapt to the new physical properties of the surrounding air. (*b*) Generally accepted tetrapodomorph phylogeny, with exemplary stem tetrapods (highlighted in light grey): (1) *Eusthenopteron*, (2) *Tiktaalik*, (3) *Acanthostega*, (4) *Ichthyostega*, (5) *Pederpes*, (6) *Greererpeton* and (7) *Eucrita* as well as exemplary early tetrapods (highlighted in dark grey) like the temnospondyls (a1) *Gerrothorax*, the embolomere (b1) *Proterogyrinus*, the microsaur (b2) *Tuditanus* and the diadectomorph (b3) *Limnoscelis.* The position of lissamphibians within temnospondyls represents the currently favoured systematic hypothesis (i.e. temnospondyl hypothesis) [1–7]. By contrast, *LH and *PH represent the alternative lepospondyl and polyphyly hypotheses, indicating that the phylogenetic position of extant amphibians is still debated [5,8,9].

In order to feed, most vertebrates must successfully overcome several developmental and evolutionary challenges associated with internal and external conditions (figure 1*a*). These challenges may include morphological changes during development, diverse food types with distinct mechanical properties and fundamentally different physical properties across the media—water and air—that vertebrates commonly inhabit.

In terms of environmental conditions, the most notable change concerns the physical differences between aquatic and terrestrial environments—water and air—in which feeding must occur [10–12]. Switching from living in one medium to the other places drastically different demands on the form and function of the feeding apparatus [13–15]. Specifically, when switching from water to air, suction-based feeding behaviours (i.e. suction feeding and intraoral hydrodynamic transport) become impossible due to the low viscosity and density of air [11,13]. In this regard, one of the most intriguing aspects of the evolution of vertebrate feeding is the grand transformation from ancestral aquatic to derived terrestrial feeding behaviours [14] due to the change in the physics of the surrounding medium and the rise of new feeding apparatus forms.

Vertebrates successfully transitioned from water to land during the Middle to Late Devonian [1,16–18]. Various evolutionary drivers and constraints, such as severe droughts, shelter and humidity under the plant canopy combined with abundant prey, have been suggested as the driving factors behind the vertebrate land invasion [16,17]. It is unlikely that the water-to-land transition was a one-off event for vertebrates; instead, it was likely completed across several amphibious, probably shore-dwelling, tetrapodomorph lineages. Thus, it remains unclear which clade gave rise to extant tetrapods, but regardless, many fossil tetrapods exhibit traits that potentially enabled semi-terrestrial life or at least brief terrestrial excursions [2,16,19,20]. Different physiological functions—including feeding—must proceed without the surrounding aqueous medium to complete the release from aquatic environments (figure 1*a*). However, the study of adaptations of these physiological functions has to date mostly been focused on the change from fins to limbs for locomotion [21,22] and from gills to lungs for respiration [23–25]. While some valuable contributions, including (functional) anatomical [26–28], suture morphology [29,30] and biomechanical studies [13,31,32], have recently drawn renewed attention to the study of feeding adaptations associated with early tetrapod water-to-land transitions, this scientific area remains poorly resolved. Here, we consider how terrestrial feeding might have evolved in vertebrates to address an essential piece of the puzzle regarding vertebrate water-to-land transitions.

## 2. Vertebrate feeding across the water-to-land transition

### (a) How to study the water-to-land transition of feeding?

The vertebrates that transitioned from water to land were tetrapodomorphs, a clade comprising crown tetrapods and their closest lobe-finned (sarcopterygian) relatives, the stem tetrapods (figures 1*b,* 2*a*). Stem tetrapods are all tetrapodomorphs more closely related to tetrapods than the ancestral sarcopterygian lungfishes (Dipnoi). Tetrapods can be further subdivided into early tetrapods and the extant groups of lissamphibians and amniotes (including lizards and their allies (sauropsids) and mammals (synapsids)). Early (crown) tetrapods belong to the stem groups of lissamphibians and amniotes, which lack derived traits that qualify them as either. The critical phylogenetic position of stem and early tetrapods and the fact that members from both groups have been suggested to have lived amphibiously [50] renders them prime candidates for studying the evolution of tetrapod feeding across the water-to-land transition.

**Figure 2.**
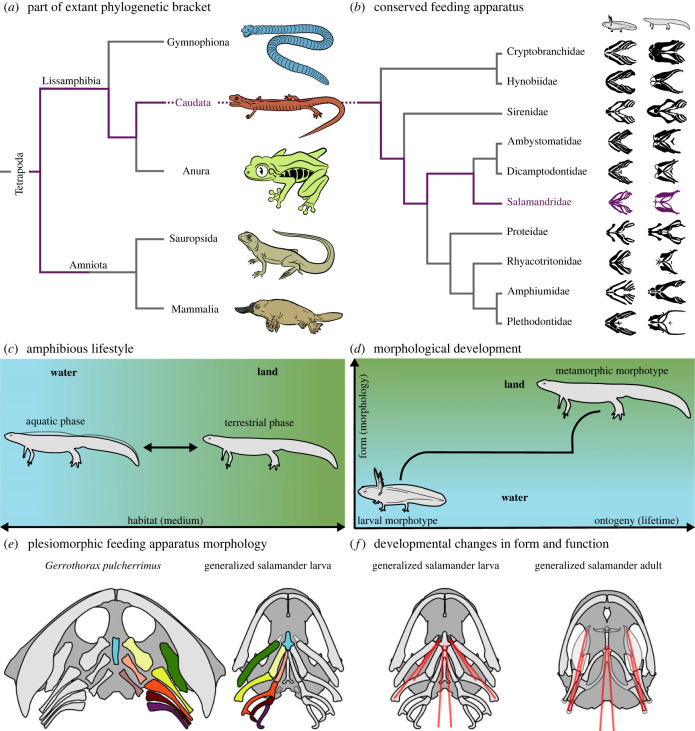
Traits rendering salamanders suitable model organisms for studying the vertebrate water–land transition. (*a*) Salamanders are extant tetrapods. (*b*) Among salamanders, the family of Salamandridae possesses a conserved feeding apparatus. The salamander phylogeny is after Pyron and Wiens [33]. The hyobranchial ontogeny was redrawn from own µCT scans and the following references: Cryptobranchidae [15,34], Hynobiidae [15,34,35], Sirenidae [34,36,37], Ambystomatidae [38,39], Dicamptodontidae [36,40], Salamandridae [39,41], Proteidae [42–44], Rhyacotritonidae [36,45], Amphiumidae [34,36,46], Plethodontidae [15,34,47]. (*c*) Some salamanders exhibit an amphibious lifestyle, freely switching between aquatic and terrestrial habitats. (*d*) Many salamanders develop from aquatic larvae to terrestrial adults (i.e. metamorphs). (*e*) Many salamanders retained plesiomorphic features of the ancestral tetrapod feeding apparatus (homologous elements are represented by the same colour), figure derived from [1,16,21,48]. Due to its particularly broad skull, *Gerrothorax* does not represent a generalized early tetrapod. Nevertheless, we selected *Gerrothorax* because of its known and relatively complete skull morphology. (*f*) The feeding apparatus of salamanders derives novelties in its form and function across metamorphosis (the red lines exemplarily display the major hyobranchial muscles of salamandrids), figure derived from [13,49].

Extant phylogenetic bracketing approaches are often used to infer the likelihood of unknown traits in organisms based on their position in a phylogenetic tree [51,52]. However, vertebrate feeding behaviours are varied and complex, and the behaviour of an extant species cannot be explained solely by its position in the phylogenetic tree nor by other known parameters like food types or the inhabited medium (water or air). This is because feeding depends strongly on the morphology of the feeding apparatus, which comprises all parts integral to the feeding behaviour of a given species (e.g. the cranium, jaws and hyobranchial structures) and the vertebrate feeding apparatus has changed dramatically throughout evolution [53–56]. These changes are unsurprising considering the major changes required for jawless, relatively small, fish-like vertebrates that initially fed using aquatic ram filtration (opportunistic feeding) to evolve into jawed, large, terrestrial predators, scavengers and grazers (targeted feeding). Therefore, we propose initially studying the form–function relationship [57] of the feeding apparatus across different environments (water and air) in relatively closely related species displaying the same general morphotype. When the form–function relationship of a morphotype is known, it may enable interpretations based on feeding apparatus morphology in taxa of similar morphology (i.e. belonging to the same general morphotype). Indeed, the century-old interest in the life-history and morphology of fossils has yielded detailed descriptions and functional interpretations [2,16], many of which are based on morpho-functional approaches, sometimes in a comparative context [58–61], and using extant vertebrate analogues for comparative approaches has emerged as a preferred way of studying the form, function and mechanical capabilities of fossil vertebrates [29,51,62,63]. However, it seems necessary to emphasize that morphology-based interpretations on feeding should be restricted to relatively closely related species of a similar morphotype or species that likely evolved convergently, displaying conform feeding apparatus morphologies across wider phylogenetic distances.

Following the detailed study of the form–function relationships, computer modelling seems particularly suited to experimentally test the resulting hypothesis about the foraging behaviour of specific fossil taxa across different environments. These approaches can enable the construction of feeding models that consider the physical conditions of water and air. These approaches may include multibody dynamics analysis (MDA), finite-element analysis (FEA) and computational fluid dynamics (CFD) to answer questions about potential biting forces, stress distributions or how liquids and foods may have been moved during feeding.

### (b) How to study vertebrate feeding?

**Figure 3.**
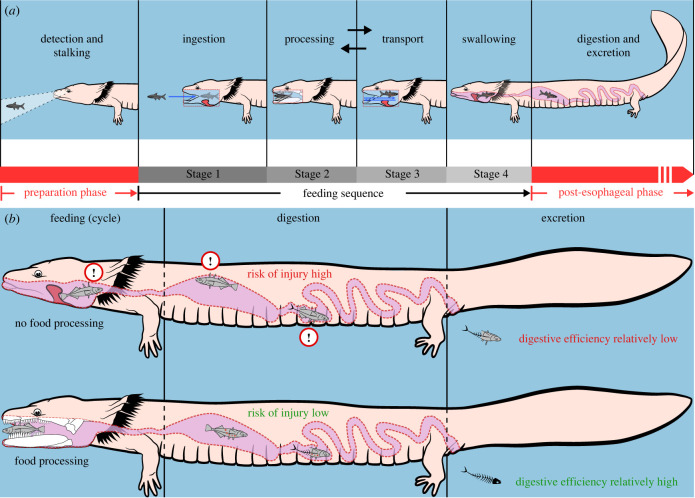
Stages of aquatic vertebrate feeding and the importance of intraoral food processing. Feeding stages and their significance are exemplarily depicted using the aquatic feeding of *Necturus maculosus*. Probably the most scientifically studied part of feeding is the so-called feeding sequence or cycle (*a*) because the feeding sequence consists of behaviours that are relatively easy to study and compare using kinematics. The feeding sequence consists of four stages: ingestion, processing, transport and swallowing of food. The blue arrows indicate the water movement during the feeding stages and the highlighted red structure outlines the oral tongue flap. (*b*) The physiological value of food processing. Note that vertebrate feeding behaviours can be much more diverse.

Vertebrate feeding proceeds in complex cycles of alternating and coordinated mechanisms [64–67]. The general mechanism of vertebrate feeding may be broken down into a preparatory phase (i.e. detection, and in predatory vertebrates, stalking), the kinematically described feeding sequence (i.e. ingestion, intraoral processing, transport and swallowing) and the post-oesophageal phase (i.e. digestion and excretion) (see the example using the feeding behaviour of *Necturus* in figure 3*a*). Comparative studies have primarily focused on the feeding sequence, which can be analysed and compared using biomechanical methods [68–70]. In most vertebrates, this feeding sequence involves the acquisition (i.e. ingestion), mechanical preparation and deterioration (i.e. processing), passing through and around within the oral cavity (i.e. transport) and pharyngeal emptying (i.e. swallowing) of the food [71]. The sequence of these steps or stages can vary, for instance, as transport and orientation of the food in the oral cavity may take place before or between individual processing bouts (see back-and-forth arrows between feeding stages 2 and 3 in figure 3*a*) depending on many factors, like the position, the processing state and the movement of the food. The anatomical and biomechanical bases of certain feeding stages may also differ across vertebrates; for example, while most vertebrates only use their tongue (or the hyobranchial system) to reposition food in the oral cavity, some fish, as well as many mammals, also use lips for the same purpose [72]. It is, therefore, important to examine the entire form–function complex over all kinematically important stages of the feeding behaviour.

It is also essential to distinguish between the stages of the feeding sequence, as they are not equally dependent on internal and external factors and conditions (e.g. the medium in which feeding takes place, the morphology of the feeding apparatus or the type of food and its mechanical properties). Distinct intraoral behaviours (e.g. rostral food transport and processing) are kinematically similar in certain salamanders, thus complicating their disambiguation (see electronic supplementary material, video S1). Therefore, in addition to kinematics, we considered the implicit behavioural intention by observing how a given behaviour affects the food in the oral cavity. Specifically, the purpose of a feeding behaviour is studied via behavioural observations focusing on the direction of movements and potential food deterioration to enable a more precise classification despite kinematic overlaps. If the food receives no or negligible damage and is gradually transported towards the GI-tract, it can be argued that transport is the most prevalent intraoral behaviour. By contrast, if the food receives more intense damage [73] and remains either relatively stationary between the jaws and or is cyclically transported back and forth between the jaws and the rest of the oral cavity, then the suite of intraoral behaviours used likely includes food processing [74]. With these distinctions in mind, we compile the findings from the past decade of research on salamander feeding and compare those results with data from unpublished studies and observations to present a refreshed model of feeding across water–land transitions. These results are then used to make inferences about the evolution of vertebrate feeding during the transition from water to land.

### (c) Modelling stem and early tetrapod behaviours

Salamanders (caudates) have become frequently studied models for stem and early tetrapods [75–78]—but why? There are limitations and challenges in palaeontology in general and in using extant species as proxies for understanding the behaviour of ancient, extinct species. These challenges and constraints include: (i) the selective preservation of fossils based on distinct taphonomic processes in different habitats; (ii) the use of simplifying assumptions (e.g. that conserved anatomical traits function the same today as they did in their ancestral state); (iii) that evidence from different approaches sometimes allow for different conclusions [79]; (iv) the primary conservation of ossified skeletal elements with information on the form and function of cartilaginous and soft tissues largely being lost; and (v) even if muscle attachments can be identified, only the largest and most superficially attached muscles can often be reconstructed. Morpho-functional interpretations in palaeontology are, therefore, always limited in precision and accuracy.

Nevertheless, amphibians in general, and salamanders in particular, are prime candidates for studying questions concerning the life and evolution of stem and early tetrapods, because: (i) as lissamphibians, they are extant tetrapods (figure 2*a*); (ii) newts (salamandrids from the subfamily *Pleurodelinae*) in particular are a clade in which the feeding mechanism had been suggested to have been partially conserved during evolution [80–82] (figure 2*b*); (iii) many salamanders exhibit an analogous lifestyle to that inferred for stem and early tetrapods, involving frequent switches between aquatic and terrestrial habits (i.e. amphibious lifestyle) [2,16] (figure 2*c*); (iv) many salamanders change from an aquatic, gill-bearing stage, via a semi-aquatic stage, to a terrestrial stage during ontogeny, similar to the proposed evolutionary trajectory of early tetrapods [83–88] (figure 2*d*); (v) salamanders often possess relatively broad and flat skulls [89,90] and a hyobranchial apparatus with an anatomy similar to that of most early tetrapods [91] at least during the early stages of larval development (figure 2*e*). Because they share many plesiomorphic traits with ancestral tetrapods, salamanders are often argued to display the least derived characters among extant lissamphibians [92–94]. Additionally, many salamanders acquire innovations in the form and function of the feeding apparatus during ontogeny, across metamorphosis and often also with a change of habitat [95–98] (figure 2*f*). This ontogenetic change in form and function of the feeding apparatus may be similar to the change that the vertebrate feeding apparatus had to undergo during the water–land transition. Therefore, salamanders seem suitable to study the evolution of feeding in stem and early tetrapods and, thus, how feeding evolved across the vertebrate water-to-land transition, similar to what has been done to reconstruct changes in locomotion across the vertebrate water-to-land transition [99–102].

Further, many stem and early tetrapods may have lived amphibious or semi-aquatic lives [2,16] similar to some extant salamanders. Newts are particularly interesting in this regard as they switch seasonally between an aquatic and a terrestrial lifestyle (i.e. multiphasic lifestyle; figure 2*c*) [84], only undergoing relatively minor morphological changes in their feeding apparatus [97,103,104]. Since newts also develop from aquatic larvae to semi-terrestrial metamorphosed adults, they allow for studying the water-to-land transition, as linked to substantial as well as minor changes in feeding apparatus morphology (i.e. metamorphosis and seasonal switches, respectively). Although salamandrids appear to be a particularly promising model taxon, it is important to consider the feeding behaviours of all salamander groups to improve our understanding of form–function relationships and, thereby, the evolution of feeding.

Consequently, the combination of their phylogenetic position and their morphology render salamanders the most suitable model organisms for reconstructing functional traits of stem and early terrestrial vertebrates. Therefore, a comprehensive overview of the ontogeny and phylogeny of salamander feeding behaviours will allow for more precise conclusions about the feeding behaviour of stem and early tetrapods. Particularly vivid and demonstrative in this regard is the possibility of experimentally simulating the Devonian vertebrate water-to-land transition *in vivo* through the lens of ontogenetic switches between aquatic and terrestrial habitats that many extant salamanders such as salamandrid newts undergo (figure 2*d*).

## 3. Salamander feeding across ontogenetic water-to-land transitions

The past decade has seen a resurgence in research on salamander feeding in general and particularly in the context of water–land transitions. This renewed focus has provided new insights into biomechanical transitions between aquatic and aerial media. A portion of these findings were already integrated into a comparative mechanical review [13]. However, we argue that our specific approach using salamanders, and new insights from the refocused area of salamander food processing, combined with more than a decade of observations of salamander feeding and as-of-yet unpublished data, warrant a renewed perspective of the function of vertebrate feeding systems during aquatic–terrestrial transitions. Our studies and observations on salamander feeding cover a broad range of species. However, to achieve clarity and comprehensibility, we limit our considerations to 40 species covering all salamander families except the Rhyacotritonidae (see electronic supplementary material, table S1). The form and function, as well as the ecology and taxonomy of salamander feeding, are summarized in table 1.

**Table 1.** Salamander feeding behaviours across phylogeny, morphotype and medium. AC, arcuate chewing; aq, as a prefix to indicate the aquatic phase in newts; Aqua, aquatic; DCC, dimensionally complex chewing; Hydro, hydrodynamic; Hyo, hyolingual; Iner, inertial; JP, jaw prehension; N/A, no answer; PS, prey shaking; RF, rotational feeding; SF, suction feeding; ter, as a prefix to indicate the terrestrial phase in newts; Terr, terrestrial; LP, lingual prehension; TPR, tongue–palate rasping. The morphotypes larval, pre-metamorphic and metamorphosed reflect the morpho-developmental state of the feeding apparatus. The pre-metamorphic morphotype displays remodelled hyobranchial structures, palatal bones and teeth as well as a slight posterior rotation of the jaw suspension and externally a more rounded snout, a larger gape and a deeper, more robust cranium (see ‘Heterochrony’). The identification of the morphotypes is based on a combination of external anatomical assessments, µCT analyses of the sampled individuals or reference material, and anatomical descriptions from the literature. While metamorphosed salamanders may amphibiously switch from aquatic-to-terrestrial environments depending on many factors, only newts show seasonal morphological changes connected to their switch from one to the other habitat. Thus, the prefix ‘aq’ and ‘ter’, which indicate a more aquatic or a more terrestrial morphotype, respectively, only apply to newts. Note that because swallowing has been suggested to be relatively uniform [34,105], has not yet been studied comparatively in salamanders, and was not externally examinable, it is not incorporated in this table. Only one metamorphic hynobiid (*Hynobius dunni*) has been studied in detail with regard to its terrestrial feeding behaviour. This videographic study suggests that *H. dunni* does not process its food. Therefore, the interpretation that metamorphic Hynobiidae may not process their food is primarily based on one species and requires further study. References used for this table include the following articles [14,46,49,73,106–125] and online videos associated with the specific behaviours in electronic supplementary material, table S1.

phylogeny	conditions		feeding behaviours				
family	morphotype	habitat	subduing^a^	ingestion	processing	transport	gulping^a^
Cryptobranchidae	pre-metamorph	Aqua	PS	SF (+ JP^a^)	AC	Hydro	Hydro
Hynobiidae	larval	Aqua	PS	SF (+ JP^a^)	DCC	Hydro	Hydro
	metamorphosed	Aqua	PS	JP^a^/SF	N/A	N/A	Hydro
	metamorphosed	Terr	PS	JP/LP	none?	Hyo	Hyo
Sirenidae	larval	Aqua	PS	SF (+ JP^a^)	DCC	Hydro	Hydro
Ambystomatidae	larval	Aqua	PS	SF (+ JP^a^)	DCC	Hydro	Hydro
	pre-metamorph	Aqua	PS	SF (+ JP^a^)	AC	Hydro	Hydro
	metamorphosed^b^	Terr	PS	JP^a^/LP	AC^b^	Hyo/Iner^a^	Hyo/Iner^a^
Dicamptodontidae	larval	Aqua	N/A	SF	N/A	N/A	N/A
	pre-metamorph	Aqua	PS	SF (+ JP^a^)	AC	Hydro	Hydro
	metamorphosed	Terr	N/A	JP^a^/LP	N/A	Hyo/Iner^a^	Hyo/Iner^a^
Salamandridae	larval	Aqua	PS	SF (+ JP^a^)	DCC	Hydro	Hydro
	pre-metamorph	Aqua	PS	SF (+ JP^a^)	AC	Hydro	N/A
	(ter) metamorphosed	Aqua	PS	SF	TPR	Hydro/Hyo	Hydro
	(ter) metamorphosed	Terr	PS	JP^a^/LP	TPR	Hyo	Hyo
	(aq) metamorphosed	Terr	PS	JP^a^	TPR	Hyo	Hyo
	(aq) metamorphosed	Aqua	PS	JP^a^/SF	TPR	Hydro/Hyo	Hydro
Proteidae	pre-metamorph	Aqua	PS	SF (+ JP^a^)	AC	Hydro	Hydro
Rhyacotritonidae	N/A	N/A	N/A	N/A	N/A	N/A	N/A
Plethodontidae	pre-metamorph	Aqua	PS	SF (+ JP^a^)	AC	Hydro	Hydro
	metamorphosed	Aqua	PS/RF	JP^a^/LP	N/A	N/A	Hyo
	metamorphosed	Terr	PS	JP^a^/LP	TPR	Hyo	Hyo
Amphiumidae	pre-metamorph	Aqua	PS/RF	SF (+ JP^a^)	AC	Hydro	Hydro

^a^Indicates the connection to prey exceeding the oral cavity.

^b^The induced metamorphosis in *Ambystoma mexicanum* results in a hyobranchial configuration that does not permit pronounced tongue projection. See electronic supplementary material, table S1 for further information (e.g. species).

### (a) When food size exceeds oral cavity capacity

Consuming large food items is common among salamanders but problematic because most salamanders do not possess the means to bite foods into pieces. Thus, large food items must be ingested whole, but it might also be necessary to prepare the food before swallowing as live prey could harm the predator from within. Seizing large prey in aquatic and terrestrial conditions often results in partial ingestion, where the food is held between the jaws. Then, struggling prey may be subdued using pronounced lateral or dorsoventral movements of the head, referred to as prey- or head-shaking [34,106,127]. Prey shaking has been suggested to be an ancestral behaviour for vertebrates [128], and the behaviour seen in salamanders seems to be similar to that reported from caecilians [129,130] and lepidosaurs [128,131,132]. This behaviour may weaken, immobilize or kill prey before complete ingestion [128,129,133]. Since subduing behaviours, such as prey shaking, serve to control prey behaviour but potentially also harm prey, they could be viewed as distinct manifestations of a continuum of pure subduing and food-processing behaviours. Interestingly, some salamanders roll (tilt) their heads to one side, scraping and squeezing the prey across the ground, as reported for caecilians [129]. While this simple behavioural change helps orient or bring the food into the mouth, it also enhances the chances of damaging the prey and, therefore, increases the processing effect from prey shaking.

Another more extreme form of subduing is related to gripping the prey using the jaws and spinning around the longitudinal axis of the body, sometimes connected with lateral shaking of the body and head, a mechanism often described as rotational feeding, twist feeding or the death roll in crocodilians [134,135]. Depending on the prey type, rotational feeding can cause significant damage and help reduce the prey prior to intraoral processing [136]. Rotational feeding sometimes helped to tear off parts of the food in salamanders but has only been observed during aquatic feeding in adult paedomorphic and metamorphosed salamanders, which may indicate that complex axial skeletal control is necessary and must be learned during ontogeny.

After being subdued and if not reduced due to either prey shaking or rotational feeding, oversized food must be forced into and through the oral, branchial and pharyngeal cavities while simultaneously being processed and swallowed because salamanders lack the dentition and musculature to enable cutting food into pieces. This behaviour results in a high energy intake per prey item and can nourish some salamander species for months. Even though gulping seems to be a prime feeding behaviour in salamanders, it is also problematic because different stages of the feeding cycle require the same underlying structures, which, thus, likely cannot perform as they usually would during the consumption of foods fitting the oral cavity. Therefore, it may be pertinent to distinguish gulping behaviours from intraoral food processing, transport or swallowing behaviours. Still, a rigorous statistical distinction of the underlying kinematics has not yet been published.

During aquatic feeding, alternating suction and jaw grasping events power gulping and gradually move the food into the mouth [105,136]. On land, a combination of inertial feeding (table 1) [128,137] and cyclic movements of the hyolingual apparatus (i.e. tongue) may carry the food posteriorly during gulping. The latter hyolingual transport mechanism involves repeated tongue protraction, slipping it underneath the food, which is retained at the palate, followed by tongue and food retraction [64,138,139]. Our observations during own studies as well as from online videos suggest that inertial feeding may be restricted to slippery or very large prey that cannot be transported using hyolingual movements alone (see electronic supplementary material, table S1). It appears that salamanders use a mix of inertial feeding and tongue-based transport, similar to many lizards [140]. During gulping, the marginal teeth of some salamanders appear to cyclically contact large partially ingested prey in a biting manner on their way into the oral cavity. Whether these behaviours represent genuine bites to damage the food (i.e. a form of processing) or merely fix and hold the prey in place during the recovery phase of the food relocation process (i.e. transport) remains unclear.

However, despite most salamanders having relatively weak jaws, amphiumas and some plethodontids can bite foods into pieces [127,136]. The form and function of chewing in plethodontids are fascinating because they possess a bilateral ligament connecting their atlas to the coronoid processes of the prearticular of their lower jaw. Stress exerted on the ligament during rapid head depressions (i.e. head tucking) causes an increase in bite force [127,141,142] and, thus, permits strong bites capable of injuring and sometimes bisecting prey [127].

### (b) When food fits within the oral cavity

#### (i) Ingestion

A feeding sequence starts with the ingestion of food, as did the founding kinematic studies on salamander feeding [38,68,143]. Although these early works evaluated kinematic differences in ingestion or food-capture behaviours and how they relate to ontogeny [38,96,144] and fluid-environmental differences [34,105], they left unaddressed some critical questions related to feeding across water-to-land transitions.

Aquatic-feeding salamanders, like many other aquatic feeders, usually apply suction feeding in which they quickly open their mouth and often simultaneously depress and retract their hyobranchial or hyolingual apparatus to expand the space of the oropharyngeal cavity and thus generate a pressure drop that induces strong water flows directed towards and through the opening mouth [145–147]. Although there are differences in performance [15,108,148], most salamanders apply similar mechanisms of suction feeding regardless of the morpho-developmental state of their feeding apparatus [98,109,149]. Available data suggest that the form of the feeding apparatus seems to be less critical in determining the mode of ingestion than the medium in which feeding occurs [98].

However, instead of the relatively consistent piston-like movement of most hyobranchial systems of salamanders to suck in food, the Chinese giant salamander *Andrias davidianus* captures its prey with an alternative mechanism. Ingestion in the Chinese giant salamander is powered by the rapid opening of its broad upper and lower jaw surfaces to accelerate the water into the mouth, while the subsequent depressing hyobranchial apparatus mainly serves to generate volume space for the inflowing water [110]. This modified suction feeding mechanism is fascinating in the context of the vertebrate water-to-land transition because many stem and early tetrapods exhibited similarly broad and flat skulls [2,16,19]. Therefore, by releasing the hyobranchial system from mechanical constraints like its primary role of suction generation, modified suction feeding behaviours may also have played a key role in the evolution of terrestrial vertebrates.

Salamandrid newts switch seasonally between aquatic and terrestrial lifestyles and need to ingest foods effectively under both conditions. Thus, newts are prime candidates for modelling food ingestion behaviours across the water-to-land transition, where the feeding apparatus morphology remains largely consistent. Newts can flexibly adjust their prey-capture behaviour to the fluid medium [103,111,150]. In aquatic conditions, newts generally use suction feeding, whereas, on land, the aquatic morphotype (aquatic staged newts) use their jaws. By contrast, the terrestrial morphotype (terrestrial staged newts) use their tongue to grasp prey (i.e. hyolingual prehension) when feeding on land. Of particular interest in this context is the Himalayan newt *Tylototriton verrucosus*, which is predominantly terrestrial and becomes aquatic only during its brief breeding season. One might assume that the Himalayan newt cannot flexibly adapt its feeding behaviour and will only catch prey on land using its tongue. However, regardless of its predominantly terrestrial lifestyle, the Himalayan newt can adjust its feeding behaviour to the aquatic condition [103]. Such flexible responses might be common in salamanders, which are primarily terrestrial but occasionally switch to aquatic life, such as, e.g. other more terrestrial newts, many ambystomatids or some plethodontids of the genus *Eurycea*. The question of why newts do not apply hyolingual prehension during their aquatic life phases was addressed in an anatomical study, which showed that when switching to water, the complex adhesive system of the newt tongue degenerates, thus precluding effective hyolingual prehension [97].

Hyolingual prehension is not unique to newts but is used in all salamander families with species of a metamorphic morphotype [105]. Particularly interesting in this context are plethodontids, which have evolved powerful ballistic tongues [151–153]. Contrary to what one would infer from observations in salamandrid newts, metamorphosed plethodontids do not use suction feeding but instead use tongue- or jaw-based prehension when feeding aquatically, whereas larval and paedomorphic plethodontids do suction feed [15,112,154]. The contrast to newts in plethodontids can be explained by their often relatively large-scaled yet delicate hyobranchial system, which fails to support the forces necessary for a powerful suction generation [15,112]. Regarding food-capture behaviours, available data thus suggest that amphibians are characterized by a relatively high degree of flexibility.

#### (ii) Transport

Transport mechanisms during feeding have often been referred to as ‘prey transport’ [105,138,139]. Further, ‘manipulation’ has been used as an umbrella term for intraoral feeding behaviours including both transport and processing [82,106]. Manipulation has been a helpful term in studies restricted to externally visible feeding behaviours when it was unclear whether the food was being processed or transported. However, because it does not provide any information on the actual feeding stage, we discourage using the term manipulation and instead support using the more specific terms transport and processing (compare 3*a*, stages 2 and 3).

Once food has been ingested, intraoral food transport usually occurs as the second or third phase of the feeding cycle (figure 3*a*). Whether transport or processing occurs first seems to depend on the position of the food in the mouth after ingestion. It further appears that transport and processing behaviours generally alternate in salamanders, which process food depending on its location in the oral cavity, its processing state, and the presence or absence of struggling motions. Despite comprehensive research on intraoral food transport in salamanders, a detailed description of transport is complicated by the fact that (i) many of these studies were based on the erroneous assumption that food size does not matter and (ii) transport mechanisms cannot always be clearly disambiguated from processing mechanisms [73,74]. The latter can be because during the intended transport of food items, these might be damaged—or the foods might be moved across the oral cavity during intended processing behaviours. Since neither the damage inflicted on prey nor its passage through the oral cavity can be exclusively attributed to food processing or food transport, these behaviours might be regarded as the two extremes of a continuum. However, our method of implying behavioural intentions based on the direction of the food movement and its processing state, as described above, allows determining if a given behaviour cycle has a more substantial transport or processing component.

#### Caudal food transport

The best-studied transport behaviour in salamanders is the caudal transport of food [105,138,155]. However, some of these studies on caudal food transport used foods that exceeded the capacity of the oral cavity of the salamander [138,139]. When feeding on large food items, several stages of the feeding cycle may be combined (i.e. ‘gulping’).

Regardless of the uncertainties associated with feeding events on relatively large foods, some important conclusions about caudal food transport can be drawn from previous studies and our observations. First, caudal aquatic food transport is achieved by hyobranchial movements, or hyolingual movements in metamorphosed salamanders, that induce water currents similar to those during suction feeding [34,137]. Second, only metamorphosed salamanders live on land and perform caudal terrestrial food transport. On land, cyclic movements of the hyolingual apparatus (i.e. the tongue) carry the food posteriorly, after which the tongue slips forward underneath the food, which is retained at the palate by its dentition [138,155,156], which stabilizes the food as the tongue moves rostro-dorsally. Our feeding observations have shown that different tongue–food–palate interactions occur during salamander feeding. While there seem to be more caudal transport behaviours during terrestrial feeding, by number, the most terrestrial tongue-food-palate interactions in salamandrids and plethodontids seem to facilitate processing. Indeed, these alleged ‘transport’ behaviours have also been suggested to enable food processing [155–157]. Because of the potential significance of food processing during many of the behaviours described as caudal food transport and their kinematic similarities to a particular type of food processing (i.e. tongue–palate rasping), we discuss these behaviours in the section ‘Processing’.

#### Rostral food transport

Rostral-directed food transport is less studied and may serve to eject potentially harmful, indigestible or distasteful food and may also be used to position the food before or during a processing cycle. Rostral positioning of food before it is processed often occurs before a series of processing bites (i.e. processing bout) or before each processing bite or strike in many aquatic suction feeders. Besides some descriptions from one species feeding in one medium [158], the kinematics of rostral-directed food transport have not been sufficiently studied across water-to-land transitions. Our unpublished observations from prior *Triturus carnifex* and *Siren intermedia nettingi* studies suggest that rostral food transport largely follows the same pattern as caudal food transport; salamanders with a larval morphotype (i.e. most larvae and some paedomorphic individuals), which only feed aquatically, seem to strongly depend on water currents generated by hyobranchial movements (i.e. hydrodynamic transportation) to move the food in the oral cavity. By contrast, salamanders that display a metamorphosed morphotype (i.e. salamanders that have undergone a metamorphosis of their hyobranchial system) use contact-based transportation where the tongue contacts the food carrying it across their oral cavity (i.e. hyolingual transport) on land. However, during aquatic feeding, metamorphosed morphotypes may apply either contact-based hyolingual or hydrodynamic transportation (or a mixture of both). The fact that metamorphosed salamanders may use a combination of hydrodynamic and contact-based transport might be explained by the fact that the tongues' adhesive system may not be sufficient to perform the terrestrial contact-based transport of food in water. However, the post-metamorphic tongue can still push the food and currents of water across the oral cavity in front of it. Consequently, and contrary to findings regarding food ingestion, both caudal and rostral hyolingual transport seem to depend more firmly on feeding apparatus morphology than on the surrounding medium. While our observations seem to provide a clearer picture, future comparative kinematic studies are needed to corroborate our predictions.

### (iii) Processing

While one key aspect of processing is always the mechanical deterioration and potential reduction of the food, the food may also be coated with mucus, triturated, immobilized or killed. Thus, intraoral food processing reduces the risk of being injured during feeding (figure 3*b*) and facilitates swallowing and digestion [71,159,160,161]. Intraoral processing behaviours require that the food fits within the oral cavity (i.e. a size of approximately one mouth width) [162]. While ontogenetic and fluid-dependent changes in salamander ingestion have been extensively studied [98,103,109,113,114, 157], similar studies concerning intraoral food processing remain less abundant.

Contrary to the long-held assumption that most salamanders do not process their food but swallow it whole and unprocessed [34,105,141], various salamanders have recently been shown to apply different mechanisms of intraoral food processing [49,73,74,115,116,163]. While some exceptional species like *Hynobius dunni* (see electronic supplementary material, table S1) seem to swallow their prey unprocessed as do most frogs [164,165], the observations presented here suggest that intraoral food processing is a basal caudate trait (table 1 and electronic supplementary material, table S1). Below we provide an overview of how intraoral food processing may be distributed across salamander species and their developmental morphotypes.

#### Tongue–palate rasping

Recently, metamorphosed newts have been shown to engage in a type of intraoral food processing that has been coined ‘tongue–palate rasping’ [49,73,116]. Tongue–palate rasping, although called differently, had previously only been reported for amniotes [166–168] and requires a highly movable, flexible and well-coordinated tongue. During tongue–palate rasping in newts, the tongue moves the food in a posterior to anterior loop-like motion across the oral cavity, thus rasping it against the palatal dentition, which consists of small needle-like teeth that pierce and tear open the prey [73]. The primary outcome of tongue–palate rasping appears to be the deterioration of the food as the food may either be moved in a loop that is stationary in the oral cavity or alternately ‘transported’ back and forth by processing behaviours with a caudal trend of the food and subsequent rostral transportation behaviours during which the food does not contact the teeth. As a result of tongue–palate rasping, the food may be punctured, lacerated or crushed [49,73]. Thus, given that the food is damaged and does not exclusively move towards the gastrointestinal system, the implicit intention of tongue–palate rasping is processing.

Newts have been demonstrated to adjust the number of processing cycles to distinct mechanical and physical properties of the food and the surrounding fluid [116]. While maggots were processed extensively, earthworm pieces of approximately the same size were barely processed. In addition, maggots coated in earthworm blood were processed similarly to earthworms, suggesting that sensory feedback such as smell and taste appear to induce flexible processing responses in addition to mechanical food properties. However, the general mechanism of food processing does not change across the different media, water and air in *T. carnifex* [116]. While food ingestion depends strongly on the surrounding fluid medium, food processing appears less medium-dependent. Further, these data do not support the hypothesis that changes in tongue and jaw coordination accompany terrestrialization [67].

Interestingly, other metamorphosed salamanders, including some plethodontids, ambystomatids and salamandrids, seem to display feeding behaviours that resemble the newt tongue–palate rasping [14,82,155,156]. Even though these studies converged on the idea that the food likely is being damaged intraorally as the tongue pushes it against the palate, they described these behaviours as food transport. However, because these studies were limited to external observations and lacked fluoroscopy data, it was impossible to distinguish intraoral transport and processing behaviours reliably. Further, those studies did not examine if the food was damaged as it moved from the oropharynx to the GI-tract. Our unpublished observations from prior *T. carnifex* and *Plethodon glutinosus* studies suggest that tongue–palate rasping often is the most frequent of intraoral behaviours in salamanders. Thus, it seems plausible that while individual food transport sequences may have been incorporated in these studies, tongue–palate rasping may have constituted most of the analysed cycles. Given the intriguing consistency between descriptions and kinematics of tongue–palate rasping and the alleged ‘transport’ of foods fitting the oral cavity (compare [14,73,116,155,156]), we argue that the studied behaviours are identical. The directional inconsistency of the power-stroke in *Salamandra salamandra* [14] may be explained by the fact that the beginning of the kinematic description had been chosen differently. There are several ways of determining the kinematic start of the behaviour because of its cyclic nature. For example, if we define the beginning of the supposed ‘terrestrial transport’ from Reilly [14] as the onset of cranial depression (at about 45 ms) the kinematic trends seem to resemble tongue–palate rasping (compare with [58]). Further, the authors noted that this behaviour adds a new prey processing mechanism. Consequently, while discussing food or ‘prey’ transport, it seems plausible that some earlier authors initially described a form of intraoral food processing [74]. This notion is supported by the fact that tongue–palate rasping functionally requires a combination of food processing and its transportation [73]. Therefore, the logical consequence is that one is likely to assume the presence of a transport behaviour if not considering food deterioration and the cyclic behaviour which does not place the food more closely to the GI-tract step by step.

Observations of prior feeding studies in *T. carnifex*, *Ichthyosaura alpestris* and *P. glutinosus* further suggest that the main differences between processing and transport behaviours seem to be whether the head is depressed as well as the general timing of the behaviour (see for example electronic supplementary material, video S1). While cranial depression and fast hyolingual protraction appear to facilitate an effective and powerful processing power-stroke, a slower hyolingual protraction and a stationary head seem to enable simple and potentially energy-saving caudal transport of the food in some newts and plethodontids as the food is not retained at the palatal dentition. However, kinematic studies of tongue–palate rasping in other salamander species need to confirm these findings. Finally, our unpublished studies and feeding observations seem to suggest that tongue–palate rasping may be more common among metamorphosed salamanders (table 1). But why is tongue–palate rasping restricted to metamorphosed salamanders?

#### Arcuate chewing

Arcuate chewing (i.e. arcilineal, orthal or vertical opening and closing of the jaws) resembles the basal ‘fish-like’ processing mechanism [169,170]. A study on the form and function of aquatic processing in pre-metamorphic versus metamorphosed newts [49] suggests that both observed morphotypes, although feeding exclusively underwater, performed different mechanisms of food processing. While the metamorphosed morphotype applied tongue–palate rasping, the pre-metamorphic morphotype exclusively used arcuate chewing bites when feeding underwater. Between each bite or a series of bites, the prey was quickly moved back and forth to reposition the food between the jaws using induced water currents. It was further demonstrated that substantial changes in the feeding apparatus, which were mainly related to the remodelling of the hyobranchial to a hyolingual system (i.e. tongue), enabled the shift in processing function [49]. These results suggest that the form of the feeding apparatus appears to represent the main factor determining the processing function.

Interestingly, previous authors seem to have described arcuate chewing behaviours [46,171] and parts thereof before, stating that the prey is moved repeatedly across the teeth by water flows, which potentially help to triturate the prey [117,171,172]. Combining these findings with our unpublished results from prior *S. salamandra* and *Ambystoma mexicanum* studies and our feeding observations (see electronic supplementary material, table S1) indicates that arcuate chewing may be shared among paedomorphic salamanders of a pre-metamorphic morphology. This assumption is further corroborated by recent findings of chewing in ambystomatids [74,162]. Therefore, the likely general presence of arcuate chewing in pre-metamorphic salamanders, and the fact that metamorphosing salamanders are likely to ontogenetically adjust their processing mechanisms from arcuate chewing to tongue–palatine rasping, may suggest that such transitions occur more frequently in salamanders whose feeding apparatuses metamorphose during ontogenesis.

#### Dimensionally complex chewing

Studies on the aquatic processing behaviour in paedomorphic sirenids found that their chewing power-stroke is dimensionally complex [115,163]. Morphological analyses revealed that their elaborate jaw joint (a hemicellar; ‘ball-socket-plane’ joint) allow for mandibular motions in sirenids with at least four degrees of freedom (i.e. pitch, yaw, sway and surge) in all three planes (i.e. the medial, transverse and coronal plane). Thus, dimensionally complex chewing contrasts with the arcuate chewing of pre-metamorphic salamanders, which is bound to one degree of freedom in one plane, namely pitch in the medial plane. What makes these studies so exciting is that the sirenid feeding apparatus largely resembles that of the early developmental stages of many other larval salamanders. These results, combined with our unpublished studies and observations from other early larval salamander species from online videos (table 1 and electronic supplementary material, table S1), seem to suggest that similar dimensionally complex chewing behaviours might be common among salamanders of an early larval morphotype. While the complex chewing behaviour of early salamander larvae potentially results from relatively unfused and flexible jaw anatomy, their presence nonetheless challenges existing general statements about complex chewing behaviours. Dimensionally complex chewing behaviours had been suggested to be mostly restricted to mammalian chewing (i.e. mastication) [160,168,173,174]. Thus, the presence of dimensionally complex chewing in salamanders seems to indicate that such chewing behaviours evolved more frequently and independently during tetrapod evolution [174]. Further, considering the morphological changes that occur during vertebrate development, these findings might suggest that changes in development could have given rise to dimensionally complex chewing.

### (iv) Swallowing

Swallowing is similar in terrestrial and aquatic salamanders [34,105,107]. It involves contraction of the transverse neck muscles and retractors bulbi, which, by retracting the eyeballs, push the prey down the oesophagus [59,175], except in those salamanders which lack this muscle (i.e. early larval and some paedomorphic forms). In addition, movements of the head and tongue raising (in adult, metamorphosed salamanders that possess tongues) may further assist swallowing. The laryngeal dilator and constrictor muscles open and close the larynx and glottis, except in salamanders that do not have lungs and therefore lack these muscles (i.e. lungless salamanders from the family Plethodontidae and clawed salamanders from the genus *Onychodactylus*) [34,176]. Finally, peristaltic contractions of the oesophageal smooth muscles propel the prey into the stomach [34].

## 4. Heterochrony: a developmental pattern that might ‘solve the riddle’

Heterochrony is an evolutionary-developmental pattern involving a change in the timing, rate or duration of developmental processes in a species relative to its ancestors or outgroup taxa [177,178]. As heterochrony results in changes to the relative time of appearance and the degree of development of characteristics that are present in other developmental stages of related organisms, it produces parallels between those stages of ontogeny and phylogeny [177]. Therefore, knowing how heterochronous changes influenced the evolution of different groups of salamanders may help unravel the mystery of the parallels between the specific developmental stages and the morpho-functionality of the feeding apparatus.

In salamanders, heterochronous changes can be recognized particularly clearly by the distinct developmental strategies and, thus, different resulting ontogenetic morphotypes [179–182]. Ancestrally, salamanders developed from an egg to an aquatic larva, onto an aquatic pre-metamorphic stage to a semi-aquatic or terrestrial metamorphosed adult (i.e. biphasic development) [85,182]. However, many salamanders evolved to become ametamorphic and do not undergo metamorphosis but retain larval traits throughout life (i.e. paedomorphic development, from an egg over a larval sometimes to a pre-metamorph morphotype). Further, some salamanders evolved developmental trajectories during which they rapidly grow metamorph characteristics and potentially even develop derived ‘peramorphic’ features (i.e. direct development from an egg to a metamorphosed morphotype).

While the morphological development (i.e. morphogenesis) of salamanders may be complex and different pathways may lead to the same morphological expressions [36,39,114], three main morphotypes with similar gross anatomies have been introduced to allow sorting salamanders according to their morpho-developmental state [36]. The distinct morphotypes resemble the different morphogenetic expressions present across different salamanders due to their distinct heterochronic developments. The morphotypes used herein (i.e. larval, pre-metamorph and metamorphosed) correspond to the morphological stages larval, mid-metamorphic and post-metamorphic from Rose and Schwarz *et al.* [36,49]. The terms have been slightly modified to be more accessible and to reflect better the fact that some salamanders may not undergo (complete) metamorphosis. The following morphological traits might characterize the morphotypes: (*larval*) broadly consistent hyobranchial morphology throughout the stage and across salamander species; (*pre-metamorphic*) remodelled hyobranchial structures, palatal bones and teeth, slight posterior rotation of the jaw suspension as well as externally a more rounded snout, a larger gape and a deeper, more robust cranium; and (*metamorphosed*) more mineralized and further remodelled hyobranchial system and externally often the resorption and reduction of the gills, labial folds, tail fin while the eyes are elevated and become larger, the limbs become stouter and the gill slits and gular fold become sealed [36].

Since species with different developmental strategies pass through analogous morphotypes [36] that produce consistent modes of functioning (table 1 and electronic supplementary material, table S1), a general pattern of salamander feeding during the ontogenetic water-to-land transition can be reconstructed (table 2). Deviations from this rule may be caused by induced developmental leaps (e.g. the use of thyroxine to induce an animal's metamorphosis). While it had been argued that *A. mexicanum* could be fully metamorphosed given the application of an appropriate hormone concentration at the correct stage [36], the induced metamorphosis often results in a deviant morphotype lacking important glands for the adhesive system of the tongue as well as often the ability for pronounced tongue projection [42,183]. Thus, as the morphology of the observed specimen had not been studied in detail, the ‘metamorphic’ *A. mexicanum* has been excluded from table 2.

**Table 2.** Salamander feeding behaviours across ontogenetic water-to-land transitions. AC, arcuate chewing; aq, as a prefix to indicate the aquatic phase in newts; Aqua, aquatic; DCC, dimensionally complex chewing; Hydro, hydrodynamic; Hyo, hyolingual; Iner, inertial; JP, jaw prehension; N/A, no answer; PS, prey shaking; RF, rotational feeding; SF, suction feeding; ter, as a prefix to indicate the terrestrial phase in newts; Terr, terrestrial; LP, lingual prehension; TPR, tongue–palate rasping. Note that because swallowing has been suggested to be relatively uniform [34,105], has not yet been studied comparatively in salamanders, and was not externally examinable, it is not incorporated in this table. Please also note that depending on the respective heterochronic form of somatic development (morphogenesis), not all salamander species go through all morphotypes and the associated stages of feeding behaviour or an ontogenetic water–land transition.

conditions		feeding behaviours				
morphotype	habitat	subduing^a^	ingestion	processing	transport	gulping^a^
larval	Aqua	PS	SF (+ JP^a^)	DCC	Hydro	Hydro
pre-metamorph	Aqua	PS/RF	SF (+ JP^a^)	AC	Hydro	Hydro
metamorphosed	Aqua	PS/RF	JP^a^/SF/LP	TPR/none?	Hydro/Hyo	Hydro/Hyo
metamorphosed	Terr	PS	JP^a^/LP	TPR/none?	Hyo/Iner^a^	Hyo/Iner^a^

^a^Indicates the connection to prey exceeding the oral cavity.

**Figure 4.**
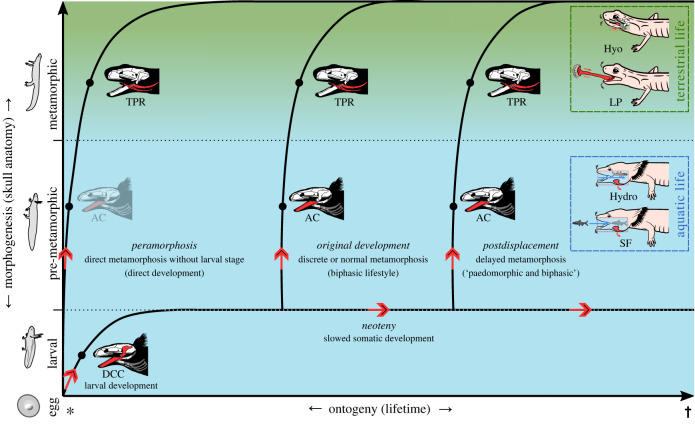
The bearing of developmental trajectories, morphogenesis and habitat on feeding in salamanders. AC, arcuate chewing; DCC, dimensionally complex chewing; Hydro, hydrodynamic food transport; Hyo, hyolingual food transport; LP, lingual prehension; SF, suction feeding; TPR, tongue–palate rasping. Note that jaw prehension and inertial feeding may be used due to properties of the prey, such as large size or mucous surface. However, jaw prehension can also be used in certain life stages with reduced lingual adhesion, as seen in newts [97,111].

**Figure 5.**
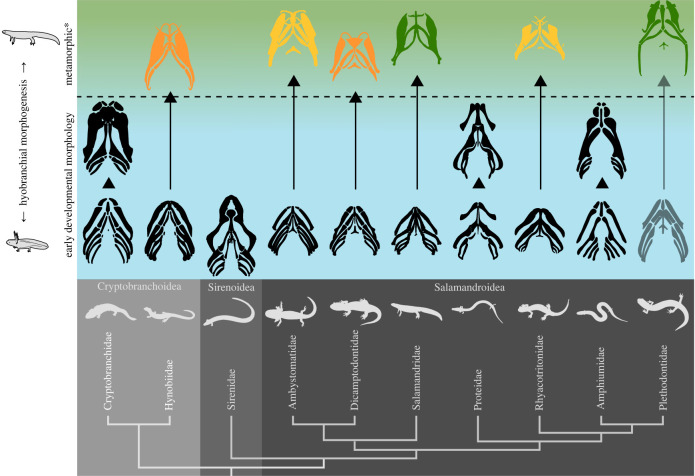
Using inferred ancestral hyobranchial morphogenesis in salamanders to predict their feeding behaviour. The dashed line represents the border for a complete metamorphosis of the hyobranchial system. The blue–green transition of the background symbolizes the transition from an aquatic (blue) to an often more amphibious lifestyle (green). The hyobranchial systems have been highlighted in: green, when they permit metamorphic feeding, including tongue prehension and tongue–palate rasping; yellow, when they are likely to permit the same general functioning; and orange, when they are less likely to enable metamorphic feeding. Hyobranchial systems painted black undergo incomplete metamorphosis and thus do not permit enhanced tongue projection and metamorphic feeding behaviours. These interpretations and classifications are based on functional–morphological considerations, including the ossification state and muscular as well as the ligamentous connection between the hyobranchial elements (of particular interest in this context is the connection between hyoid and branchial arches). However, considering that certain salamanders do not process their food at all, it seems plausible that other factors also influence the application of certain feeding behaviours. Therefore, even if salamanders have metamorphic tongues, metamorphic feeding may not be guaranteed, and further studies are needed to verify this general form–function relationship. Phylogeny after Pyron and Wiens [33]; suborders are framed with shades of grey. Hyobranchial development after numerous authors [15,34–37,39,40,42–47,144,180,182,184–190] as well as own µCT scans. Please note that the figure was created using representative species from each family that exhibit hyobranchial ontogeny, which we hypothesise are closest to the ancestral state of this family. The larval hyobranchial system in plethodontids is displayed in bright grey to signal that larval and pre-metamorphic morphotypes are uncommon in this family. The asterisk indicates that the hyobranchial morphogenetic state may also be peramorphic.

Figure 4 shows how distinct developmental trajectories lead to different morphotypes, how these distribute across water and air, and which feeding behaviours relate to these factors. The most significant metamorphic changes in the feeding apparatus concern the hyobranchial system, which is particularly important for metamorphic tongue-based feeding behaviours like lingual prehension or tongue–palate rasping. Therefore, the different morphotypes and the gross morphology of the hyobranchial system might help predict metamorphic feeding behaviours across salamanders (figure 5).

Since the hyobranchial ontogeny may be modified across species within a given family, the figure only provides an overview of the relatively basal form and its possible functioning of the hyobranchial system across salamander families. This prediction may not fit all members of each family. A good indicator of a complete metamorphic feeding behaviour might be the presence of tongue prehension and a longitudinal array of teeth on the palate because if the tongue can be extended out of the mouth, it can also perform tongue–palate interactions, and palatal teeth help damage the food.

## 5. Conclusion on vertebrate feeding across the water-to-land transition

Considering the fundamental mechanical issues of ancestrally aquatic vertebrates, scientists have suggested that acquiring a movable neck and a muscular and mobile tongue can be regarded as licences or innovations enabling feeding under terrestrial conditions [13]. Therefore, a movable neck and a muscular and mobile tongue might be key factors in colonising terrestrial environments permanently. While the development of a defined neck area is traceable across stem and early tetrapods [16,48,191], the evolution of the hyolingual system leading to the emergence of muscular and adhesive tongues remains unknown [13,192]. Given the current palaeontological record, it seems plausible to assume that flexible hyolingual systems (i.e. tongues) and, thus, hyolingual feeding behaviours evolved late and independently in stem amphibians and stem amniotes.

While it is well-known that ancestrally aquatic vertebrates, with their relatively massive and inflexible hyobranchial systems, likely switched from suction feeding to jaw prehension when venturing onto land [13,89,193,194], it remained largely speculative how they may have processed and transported food once ingested. The generalised adult anatomy of the stem and early tetrapod feeding apparatus [60,91] is consistent in many features with many salamanders' larval and pre-metamorphic morphological states. Such consistent features can be found across digitated tetrapodomorphs such as *Acanthostega* and *Ichthyostega*, but also for embolomeres (anthracosaurs), baphetids, colosteids, whatcheeriids and the majority of temnospondyls [2,90]. From this perspective, the feeding apparatuses of most early tetrapods appear paedomorphic with respect to metamorphosed salamanders. However, there was a continuity of the early tetrapodomorph condition well into early tetrapods, simply because most of these taxa likely were aquatic predators throughout their life cycles [90]. This is also apparent from their possession of open gill slits (colosteids, temnospondyls), well-ossified larval hyobranchial skeletons in adults (many temnospondyls) and the preservation of aquatic prey in their intestines (many temnospondyls). Therefore, the metamorphosed feeding apparatus of salamanders and frogs, as well as the adult amniote condition, represent evolutionarily novel, peramorphic conditions with respect to tetrapodomorphs and early tetrapods.

Generally, there seems to be broad agreement that the ancestral feeding of tetrapodomorphs occurred under aquatic conditions and that water currents were used for caudal and rostral hydrodynamic transport. At the same time, the jaws were likely used to perform chewing bites. Integrating the morphological patterns with the functional observations summarised herein, the initial terrestrial feeding of tetrapodomorphs might have been as follows: either (i) they grasped food using their jaws and dragged it back into the water (as suggested from Reilly [179]) where they may have employed caudal and rostral hydrodynamic transport, interspersed with chewing bites depending on the type of prey (terrestrial prey shaking and simple biting behaviours or aquatic rotational feeding may have preceded potential aquatic chewing); or (ii) they directly applied a combination of prey shaking and biting behaviours on the partially ingested foods, which were finally transported to the oesophagus using inertial transport (i.e. quick forward movements or head rotations while temporarily releasing the grip on the food). Although the second scenario is currently less supported since inertial transport has only been observed in metamorphosed salamanders, it still seems no less plausible than the first scenario, as it does not require a specific anatomical set-up. The feeding behaviour of early tetrapods displaying abnormal morphologies might be studied in detail using more specific lissamphibian models.

These interpretations may not constitute a major revelation for those acquainted with the evolutionary dynamics of vertebrate feeding systems. However, while adult tetrapodomorphs likely applied simple arcuate chewing bites, they may also have used dimensionally complex chewing during earlier developmental stages. Indeed, the cranio-muscular anatomy has been suggested to be preadapted for more dimensionally complex mandible movements already in stem tetrapods [160,195]. The only factor that might restrict dimensionally complex movements of the mandible appears to be the articular surface of the jaw joint [160] or mandibular suspension. We, therefore, encourage the study of early tetrapodomorph jaw articulations and suspensions—morphological details that besides very few considerations [196–198] seem to have largely been ignored. Comparative analyses of fossilised and extant articular surfaces across larval tetrapods might answer whether they used complex chewing mechanics during ontogeny. Further, the comparative study of ontogenetic changes in the jaw joint anatomy of early tetrapods might shed light on how heterochronic changes may have influenced the rise of dimensionally complex chewing.

## Data Availability

The data are provided in electronic supplementary material [199].
